# Pediatric surgical site infections in 287 hospitals in the United States, 2015–2018

**DOI:** 10.1017/ice.2022.154

**Published:** 2023-06

**Authors:** Roshni Mathew, Jorge L. Salinas, Heather E. Hsu, Robert Jin, Chanu Rhee, Grace M. Lee

**Affiliations:** 1 Department of Pediatrics, Stanford University School of Medicine, Palo Alto, California; 2 Department of Medicine, Stanford University School of Medicine, Stanford, California; 3 Department of Pediatrics, Boston University School of Medicine, Boston, Massachusetts; 4 Department of Population Medicine, Harvard Pilgrim Health Care Institute and Harvard Medical School, Boston, Massachusetts; 5 Department of Medicine, Brigham and Women’s Hospital, Boston, Massachusetts

## Abstract

Among 287 US hospitals reporting data between 2015 and 2018, annual pediatric surgical site infection (SSI) rates ranged from 0% for gallbladder to 10.4% for colon surgeries. Colon, spinal fusion, and small-bowel SSI rates did not decrease with greater surgical volumes in contrast to appendix and ventricular-shunt SSI rates.

Surgical site infection (SSI) is one of the most common and costly healthcare-associated infections (HAIs) with an estimated annual cost of >3 billion dollars in the United States.^1^ More than 200,000 pediatric surgical procedures are performed annually in the United States, of which ∼40% are performed in general acute-care hospitals.^2^ However, limited US data are available describing SSI rates in children. We describe the epidemiology of SSIs for 6 common procedures among a large cohort of US hospitals caring for pediatric patients.

## Methods

We included hospitals participating in the Preventing Avoidable Infectious Complications by Adjusting Payment (PAICAP) study that focused on evaluating the impact of financial policies on infection rates in the National Healthcare Safety Network (NHSN).^3^ We identified 287 hospitals that reported SSI surveillance data on ≥1 operative procedure in pediatric patients. Because enrollment in PAICAP was focused on Centers for Medicare and Medicaid Services (CMS) payment policies, nearly all hospitals in PAICAP are general hospitals caring for pediatric patients, and only a few are freestanding children’s hospitals. We assessed inpatient procedures in patients <18 years of age included in the Pediatric Complex Admission/Readmission (A/R) SSI Model using data from 2015–2018. We used 2017 NHSN definitions for the following surgical procedures most frequently reported to the NHSN: colon, appendix, spinal fusion, ventricular-shunt, small-bowel, and gallbladder surgeries. Complex SSI models included deep and organ space but excluded superficial SSIs (Supplementary Material 1 online).

We obtained hospital characteristics from the American Hospital Association (AHA) 2017 Annual Survey, including region, location (metropolitan, with a core urban area ≥50,000 population; micropolitan, with a core urban area ≥10,000–50,000 population; or rural), hospital bed size (small, with <100 beds; medium, with 100–399 beds; or large, with ≥400 beds), type of ownership (public, for profit, or not for profit), and teaching status (major, graduate, limited, or nonteaching) (Supplementary Material 2 online).

We have described the following procedural characteristics: American Society of Anesthesiologists (ASA) score, presence of diabetes, operative procedure duration, urgency, and wound class. We calculated (1) annual SSI rates (number of complex infections per 100 procedures) and (2) average yearly standardized infection ratio (SIR) for individual hospitals by procedure type, over time, and by procedural volume. All analyses were performed in SAS version 9.4 software (Cary, NC). The Harvard Pilgrim Health Care and Stanford University School of Medicine Institutional Review Boards approved this study.

## Results

PAICAP hospitals (n = 287) performing surgical procedures on pediatric patients were more likely to be larger (bed size, ≥400 beds), teaching hospitals, not for profit, located in metropolitan areas. They were also more likely to have neonatal and pediatric intensive care beds compared to all AHA hospitals (Supplementary Material 2 online). Most hospitals reported on colon surgery (79%). Fewer hospitals caring for pediatric patients (<25%) reported on appendix, spinal-fusion, ventricular-shunt, small-bowel, or gallbladder surgeries (Supplementary Material 2 online). Overall, 27,433 surgical procedures were included in this study: appendix surgery (41%), colon surgery (23%), spinal-fusion surgery (17%), ventricular-shunt surgery (9%), small-bowel surgery (6%), and gallbladder surgery (4%). We examined characteristics of these 6 commonly performed procedures (Table 1). Higher ASA scores were observed for colon, ventricular-shunt, and small-bowel procedures. Spinal-fusion surgeries had the longest median duration, followed by colon, and small-bowel surgeries.

**Table 1.** Characteristics of Common Inpatient Procedures Performed in Children (<18 years) Included in the All Complex Admission/Readmission Surgical Site Infection (SSI) Model, 287 US Hospitals, 2015–2018.

Operative Procedure	No. of Procedures Performed	ASA Score; Mean/Median (Range)^a^	Diabetes (% Yes)	Duration of Operative Procedure, Mean hours/Median hours (Range)	Emergent/Urgent (% Yes)	Wound Class, %			
						C	CC	CO	D
Appendix surgery	11,364	1.4/1 (1–5)	0.5	0.8/0.7 (0.1–3.5)	50.5	0.0	66.0	22.2	11.8
Colon surgery	6,364	2.6/3 (1–5)	1.2	2.6/2.2 (0.1–11.6)	23.1	0.0	68.9	20.3	10.7
Spinal fusion^b^	4,557	2.1/2 (1–4)	0.6	4.9/4.6 (0.2–14.1)	3.6	99.1	0.6	0.1	0.1
Ventricular shunt^b^	2,485	2.9/3 (1–5)	0.6	1.3/1.0 (0.1–6.3)	21.4	94.0	3.7	1.0	1.4
Small bowel surgery	1,568	2.7/3 (1–5)	0.5	2.5/2.1 (0.1–14.1)	21.0	0.0	79.5	13.9	6.6
Gallbladder surgery	1,095	1.9/2 (1–4)	1.3	1.4/1.2 (0.1–5.8)	13.3	0.0	88.6	10.9	0.5

Note. C, clean; CC, clean-contaminated; CO, contaminated; D, dirty.

a Scores 1–5; 6 is excluded.

b 90-day surveillance window for SSI (vs 30-day surveillance window for all other outcomes)

SSI rates by procedure type and volume are presented in Table 2. Surgical volumes are categorized by tertiles given the small volumes for certain procedures performed in pediatric patients. The average annual SSI rates were highest for colon surgery (5.3%−10.4%) and lowest for gallbladder surgery (0.0%−0.2%). For colon, small-bowel, and spinal-fusion surgeries, higher SSI rates were observed in hospitals with greater procedural volume (eg, colon surgery SSI rate of 8.4% for the highest volume tertile and 6.1% for the lowest volume tertile). In contrast, SSI rates for appendix surgery were lower in hospitals with higher volumes (ie, 4.3% in the lowest tertile and 1.9% in the highest tertile). A similar trend was noted for ventricular shunt surgeries (ie, 6.4% in the lowest tertile and 4.0% in the highest tertile). SIRs remained relatively stable, yet observed events were substantially higher than predicted (SIR>1) (Supplementary Material 3 online).

**Table 2.** Average Annual Surgical Site Infections (SSI) per 100 Procedures by Procedure Type and Procedural Volume, 2015–2018^a^

	Average Annual SSI Rate (No. of Unique Hospitals)					
Variable	Appendix Surgery	Colon Surgery	Spinal Fusion Surgery	Ventricular Shunt Surgery	Small Bowel Surgery	Gallbladder Surgery
**Average annual SSI rate, by year**						
2015	1.7 (48)	6.2 (153)	1.6 (41)	3.1 (11)	3.5 (22)	0.2 (36)
2016	3.7 (53)	7.9 (141)	2.2 (41)	1.8 (12)	4.0 (22)	0.0 (33)
2017	1.5 (55)	5.3 (145)	3.8 (55)	2.8 (18)	5.1 (20)	0.0 (34)
2018	2.9 (52)	10.4 (132)	2.4 (53)	9.2 (19)	2.2 (20)	0.0 (34)
**Average annual SSI rate, by procedural volume by tertile** ^**b**^						
Lowest tertile	4.3 (21)	6.1 (153)	1.3 (34)	6.4 (7)	0.0 (21)	0.0 (24)
Middle tertile	1.5 (20)	8.9 (4)	2.5 (15)	4.0 (6)	8.3 (2)	0.0 (7)
Highest tertile	1.9 (22)	8.4 (85)	3.8 (26)	4.0 (8)	6.2 (12)	0.1 (14)

Note. SSI, surgical site infection.

a Procedural volume in 2018.

b Tertiles for procedures by volume: Appendix surgery ≤9.0, >9.0 and <34.0, >=34.0; colon surgery ≤4.0, >4.0 and <6.0, ≥6.0; spinal fusion surgery ≤4.0, >4.0 and <13.0, ≥13.0; ventricular shunt surgery ≤7.0, >7.0 and <27.0, ≥27.0; small bowel surgery ≤4.0, >4.0 and <8.0, ≥8.0; gallbladder surgery <4.0, >4.0 and <6.7, ≥6.7.

## Discussion

We describe pediatric surgeries and SSI rates for 6 common surgical procedures in a large cohort of US hospitals from 2015 to 2018. SSI rates for colon, small bowel, and spinal-fusion surgeries were relatively stable or showed a modest increase in relationship to surgical volumes, which differs from the expected higher volume–better outcomes relationship.^4^ In contrast, appendix and ventricular-shunt surgeries appeared to have lower SSI rates with higher surgical volumes. Our findings are consistent with a prior systematic review that demonstrated an association between higher volume and improved outcomes for appendicitis and ventricular-shunt surgeries in children, as well as mixed findings for procedures that are less common, more resource intensive or more likely to occur in tertiary-care centers.^5^ Our data reflect the challenges with measuring quality in pediatrics; surgical volume and procedural complexity are often correlated and current metrics do not adequately capture this complexity.

The highest SSI rates were observed following colon surgery, which parallels the extant adult SSI literature on colorectal procedures associated with high SSI rates and substantial variability (3%–30%).^6^ Comparable pediatric data using NHSN definitions are scarce. However, our data are similar to those reported by the Pediatric National Surgical Quality Improvement Program (NSQIP-P), which described an overall SSI rate for colorectal procedures of 5.9% with higher rates (11.4%) for total abdominal colectomy in a cohort of 50 hospitals.^7^ Unlike our experience with other pediatric HAIs in a similar study population, we did not observe a consistent downward trend in pediatric SSI rates over the study period.^8,9^ We also did not see improvements in SIRs for any of the procedures (except gallbladder surgery) over the 4-year period of this study (Supplementary Material 3 online). The high SIRs noted in this study may be due to the differences in patient and surgical characteristics in our cohort compared to the national aggregate. Interpreting these findings is difficult, given challenges in appropriate risk adjustment for pediatric SSI models (Supplementary Material 1 online). For example, the current SIR model for ventricular shunt surgery only includes age, and the SIR model for spinal fusion does not include surgical indications such as neuromuscular versus idiopathic scoliosis.^10^ Assessing quality of care in surgical patients in pediatrics and benchmarking may require adjustment for common pediatric comorbidities that have an impact on outcomes.

Our study has several limitations. We did not have granular data on individual risk factors, which may have further informed the differences in rates across varied settings. Participating hospitals tended to be larger, metropolitan, academic institutions and may not fully represent the spectrum of pediatric care in the United States. In addition, accurate comparisons are challenging for pediatric procedures because the risk adjustment models used by NHSN for children are often limited. We did not include superficial SSIs; however, they carry lower morbidity and mortality, and they are currently excluded from the CMS benchmarking for adult SSIs. Also, we did not validate the surveillance approaches used by various healthcare facilities, which may have affected surveillance and reporting.

This study is one of the largest on pediatric SSI rates reported to the NHSN; further research on pediatric SSI measurement and prevention efforts is needed.^11^ Harmonization of criteria across different entities (NHSN, NSQIP, etc) to allow for reduced reporting burden and capture of key improvement opportunities should also be addressed. Benchmarking targets may be challenging; thus, an initial focus on common procedures such as appendix or colon surgery is warranted. Continued efforts are needed to refine and validate risk adjustment models and to promote actionable improvements in care.

## Supplementary material

For supplementary material accompanying this paper visit https://doi.org/10.1017/ice.2022.154. click here to view supplementary material
