# Supplementary material for: Pediatric surgical site infections in 287 hospitals in the United States, 2015–2018
Source: Infect Control Hosp Epidemiol. 2022 Jul 8;44(6):968–70. doi: 10.1017/ice.2022.154 (PMC10111852; doi:10.1017/ice.2022.154)
Supplement: Supplementary file 1 [file S0899823X22001544sup.zip › S0899823X22001544sup001.docx]

Supplement 3. Average yearly standardized infection ratio (SIR) by procedure type, time, and procedural volume*, 2015-2018.

|  | | **Appendix surgery** | | **Colon surgery** | | **Spinal fusion surgery** | | **Ventricular shunt surgery** | | **Small bowel surgery** | | **Gall bladder surgery** | | |
| --- | --- | --- | --- | --- | --- | --- | --- | --- | --- | --- | --- | --- | --- | --- |
|  |  | Number of unique hospitals | Avg pooled SIR per year | Number of unique hospitals | Avg pooled SIR per year | Number of unique hospitals | Avg pooled SIR per year | Number of unique hospitals | Avg pooled SIR per year | Number of unique hospitals | Avg pooled SIR per year | Number of unique hospitals | Avg pooled SIR per year | |
| Average annual SIR, by year | 2015 | 48 | 4.6 | 153 | 3.2 | 41 | 3.6 | 11 | 2.2 | 22 | 7.4 | 36 | 5.3 | |
|  | 2016 | 53 | 5.6 | 141 | 5.2 | 41 | 1.8 | 12 | 1.1 | 22 | 5.4 | 33 | 0.0 | |
|  | 2017 | 55 | 8.6 | 145 | 3.8 | 55 | 3.1 | 18 | 2.1 | 20 | 9.4 | 34 | 0.0 | |
|  | 2018 | 52 | 6.2 | 132 | 4.1 | 53 | 3.4 | 19 | 1.8 | 20 | 6.3 | 34 | 0.0 | |
| Average annual SIR by procedural volume by tertiles^+^ | Lowest tertile | 21 | 10.2 | 153 | 2.4 | 34 | 2.7 | 7 | 2.0 | 21 | 0 | 24 | | 0.0 |
|  | Middle tertile | 20 | 6.2 | 4 | 2.9 | 15 | 4.4 | 6 | 2.5 | 2 | 7.9 | 7 | | 0.0 |
|  | Highest tertile | 22 | 6.3 | 85 | 4.3 | 26 | 2.9 | 8 | 1.7 | 12 | 7.4 | 14 | | 1.7 |

*Procedural volume in 2018

^+^Tertiles for procedures by volume: Appendix surgery <=9.0, >9.0 and <34.0, >=34.0; colon surgery <=4.0, >4.0 and <6.0, >=6.0; spinal fusion surgery <=4.0, >4.0 and <13.0, >=13.0; ventricular shunt surgery <=7.0, >7.0 and <27.0, >=27.0; small bowel surgery <=4.0, >4.0 and <8.0, >=8.0; gallbladder surgery <4.0, >4.0 and <6.7, >=6.7.
