# Supplementary material for: Pediatric surgical site infections in 287 hospitals in the United States, 2015–2018
Source: Infect Control Hosp Epidemiol. 2022 Jul 8;44(6):968–70. doi: 10.1017/ice.2022.154 (PMC10111852; doi:10.1017/ice.2022.154)
Supplement: Supplementary file 1 [file S0899823X22001544sup.zip › S0899823X22001544sup002.docx]

**Supplement 1:**

The Pediatric Complex Admission/Readmission (A/R) SSI Model excludes outpatient procedures or procedures missing information on gender, closure technique, or American Society of Anesthesiologist (ASA) score. Appendix surgery, gallbladder surgery, colon surgery, and small bowel surgery were subject to surveillance for a 30-day period. Surveillance for spinal fusions, and ventricular shunts used a 90-day post-procedure period.

For the Pediatric Complex A/R SSI Model, we included organ space and deep incisional SSIs detected at the same facility where the procedure occurred. Superficial SSIs were excluded. Our outcomes of interest included crude SSI rates (number of SSIs per 100 procedures) as well as standardized infection ratios (SIR), which account for patient or procedural factors associated with infection after surgery. Procedure-specific SIRs were calculated by dividing the number of observed infections by the number of predicted infections; however, SIRs were only calculated if the number of predict infections in a given quarter was >1. Our rationale for including both outcomes was that SIRs were calculated using 2015 re-baseline data from January 2017 onward, whereas prior SIRs were based on 2006−08 baseline data. Additionally, infections present at the time of surgery (PATOS) were excluded from the numerator after January 2017.

References:

<https://www.cdc.gov/nhsn/pdfs/rebaseline/faq-ssi-rebaseline.pdf>

<https://www.cdc.gov/nhsn/pdfs/pscmanual/9pscssicurrent.pdf>

<https://www.cdc.gov/nhsn/pdfs/ps-analysis-resources/nhsn-sir-guide.pdf>
