# Supplementary material for: Pediatric surgical site infections in 287 hospitals in the United States, 2015–2018
Source: Infect Control Hosp Epidemiol. 2022 Jul 8;44(6):968–70. doi: 10.1017/ice.2022.154 (PMC10111852; doi:10.1017/ice.2022.154)
Supplement: Supplementary file 1 [file S0899823X22001544sup.zip › S0899823X22001544sup003.docx]

**Supplement 2:**

Characteristics of Preventing Avoidable Infectious Complications by Adjusting Payment (PAICAP) hospitals caring for children (complex SSI population), 2015–2018 vs. all American Hospital Association (AHA) hospitals reporting in 2017

|  | **PAICAP Hospitals (2015-2018)**  **N=287** | | **AHA Hospitals (2017)**  **N=6,210** | | p-value |
| --- | --- | --- | --- | --- | --- |
| **Characteristics** | **N** | **%** | **N** | **%** |  |
| Region, No. (%) |  |  |  |  | p<0.0001 |
| Midwest | 65 | 23% | 1706 | 27% |  |
| Northeast | 100 | 35% | 810 | 13% |  |
| South | 53 | 18% | 2504 | 40% |  |
| West | 69 | 24% | 1190 | 19% |  |
| Location, No. (%) |  |  |  |  | p<0.0001 |
| Metropolitan | 260 | 91% | 4192 | 68% |  |
| Micropolitan | 23 | 8% | 887 | 14% |  |
| Rural | 4 | 1% | 1131 | 18% |  |
| Total hospital bed Size, No. (%) |  |  |  |  | p<0.0001 |
| <100 | 28 | 10% | 3447 | 56% |  |
| 100-399 | 149 | 52% | 2246 | 36% |  |
| ≥400 | 110 | 38% | 517 | 8% |  |
| Total neonatal and pediatric beds Size, No. (%) |  |  |  |  | p<0.0001 |
| ≤25 | 151 | 53% | 3683 | 59% |  |
| 26-≤50 | 63 | 22% | 292 | 5% |  |
| >50 | 72 | 25% | 305 | 5% |  |
| Unknown | 1 | 0% | 1930 | 31% |  |
| Children’s hospital** | 6 | 2% | 100 | 2% | 0.043 |
| Presence of pediatric ICU services | 81 | 28% | 397 | 6% | p<0.0001 |
| Presence of level III/IV NICU services | 177 | 62% | 924 | 15% | p<0.0001 |
| Presence of level II NICU services | 118 | 41% | 678 | 11% | p<0.0001 |
| Type of ownership, No. (%) |  |  |  |  | p<0.0001 |
| Federal | 0 | 0% | 208 | 3% |  |
| For-profit | 30 | 10% | 1650 | 27% |  |
| Not-for-profit | 233 | 81% | 3133 | 50% |  |
| Public | 24 | 8% | 1219 | 20% |  |
| Teaching status, No. (%)* |  |  |  |  | p<0.0001 |
| Graduate | 131 | 46% | 1688 | 27% |  |
| Major | 78 | 27% | 301 | 5% |  |
| Minor | 11 | 4% | 339 | 5% |  |
| Non-teaching | 67 | 23% | 3882 | 63% |  |
| Procedure type reported in NHSN |  |  |  |  |  |
| Appendix surgery | 59 | 21% | - | - |  |
| Colon surgery | 226 | 79% | - | - |  |
| Spinal fusion | 69 | 24% | - | - |  |
| Ventricular shunt surgery | 19 | 7% | - | - |  |
| Small bowel surgery | 32 | 11% | - | - |  |
| Gallbladder surgery | 42 | 15% | - | - |  |

^*^All hospitals were placed into 1 of 4 categories based on their response to the AHA survey: major teaching hospitals (those that are members of the Council of Teaching Hospitals [COTH]), graduate teaching hospitals (non-COTH members with a residency training program approved by the Accreditation Council for Graduate Medical Education), minor teaching hospitals (non-COTH members with a medical school affiliation reported to the American Medical Association), and nonteaching hospitals (all other institutions).

^**^A children’s hospital was defined as a hospital that reported that it restricts admissions primarily to children in the AHA survey. (radmchi=1)
